# Rapid Enhancement of Subcortical Neural Responses to Sine-Wave Speech

**DOI:** 10.3389/fnins.2021.747303

**Published:** 2021-12-20

**Authors:** Fan-Yin Cheng, Can Xu, Lisa Gold, Spencer Smith

**Affiliations:** Department of Speech, Language, and Hearing Sciences, University of Texas at Austin, Austin, TX, United States

**Keywords:** frequency following response (FFR), efferent, top-down, sine-wave speech perception, auditory learning

## Abstract

The efferent auditory nervous system may be a potent force in shaping how the brain responds to behaviorally significant sounds. Previous human experiments using the frequency following response (FFR) have shown efferent-induced modulation of subcortical auditory function online and over short- and long-term time scales; however, a contemporary understanding of FFR generation presents new questions about whether previous effects were constrained solely to the auditory subcortex. The present experiment used sine-wave speech (SWS), an acoustically-sparse stimulus in which dynamic pure tones represent speech formant contours, to evoke FFR_SWS_. Due to the higher stimulus frequencies used in SWS, this approach biased neural responses toward brainstem generators and allowed for three stimuli (/bɔ/, /bu/, and /bo/) to be used to evoke FFR_SWS_ *before* and *after* listeners in a training group were made aware that they were hearing a degraded speech stimulus. All SWS stimuli were rapidly perceived as speech when presented with a SWS carrier phrase, and average token identification reached ceiling performance during a perceptual training phase. Compared to a control group which remained naïve throughout the experiment, training group FFR_SWS_ amplitudes were enhanced post-training for each stimulus. Further, linear support vector machine classification of training group FFR_SWS_ significantly improved post-training compared to the control group, indicating that training-induced neural enhancements were sufficient to bolster machine learning classification accuracy. These results suggest that the efferent auditory system may rapidly modulate auditory brainstem representation of sounds depending on their context and perception as non-speech or speech.

## Introduction

The mammalian auditory system contains extensive efferent innervation descending from the cortex to subcortex and inner ear (Winer, 2005). Numerous animal modeling studies suggest that these projections facilitate neuroplastic functional changes on multiple time scales and at multiple levels of the subcortical auditory system. For example, *online* modulation of auditory function has been observed at the level of the cochlea (Xiao and Suga, 2002; May et al., 2004; Dragicevic et al., 2015; Terreros and Delano, 2015; Delano and Elgoyhen, 2016; Lauer et al., 2021), cochlear nucleus (Hernandez-Peon et al., 1956), and inferior colliculus (Slee and David, 2015; Shaheen et al., 2021). *Short-* and *long-term* training also alters physiologic function in the same structures (Gao and Suga, 1998, 2000; Yan and Suga, 1998, 1999; Suga et al., 2000, 2002; Ji et al., 2001; Ma and Suga, 2001; Yan et al., 2005; Malmierca et al., 2009). Inversely, obliterating or temporarily silencing corticofugal efferent connections disrupts online modulation and short- and long-term training effects measured subcortically (e.g., Bajo et al., 2010; León et al., 2012). Together, these studies suggest that efferent activity is a potent force in shaping how the nervous system responds to behaviorally significant sounds, even at the earliest stages of auditory processing.

Efferent-induced changes in human subcortical auditory function have, by necessity, almost exclusively been assessed through non-invasive objective measurements. Some reports have demonstrated that otoacoustic emissions (i.e., proxy measures of outer hair cell function) are modulated online by attention (Wittekindt et al., 2014; Smith and Cone, 2015; Hernandez-Perez et al., 2021) or through short- or long-term training (Perrot et al., 2006; de Boer and Thornton, 2008; Bidelman et al., 2014, 2016, 2017). Other reports using similar methodologies have failed to replicate these findings (Stuart and Butler, 2012; Francis et al., 2018; Jedrzejczak et al., 2020). A variety of electrophysiologic measures has been used to study online or training-based neuroplastic functional changes in the human auditory subcortex including the auditory brainstem response (ABR) and frequency following response (FFR). As a general principle, the “classic” ABR does not appear to be altered by attention (Picton and Hillyard, 1974; Woldorff et al., 1987; Connolly et al., 1989; Gregory et al., 1989; Hackley et al., 1990), whereas the FFR literature presents a less cohesive narrative. Seminal work by Galbraith and Arroyo (1993) and Galbraith et al. (1995, 1998, 2003) suggested that FFRs to simple (e.g., tonal) and complex (e.g., dichotic speech) stimuli were modulated during auditory or visual attention. While some researchers have replicated these findings (e.g., Hairston et al., 2013; Lehmann and Schönwiesner, 2014), others have failed to observe attention effects and have questioned whether previous results were influenced by task-based differences in FFR residual noise (Ruggles et al., 2012; Varghese et al., 2015). More recent studies demonstrate FFR enhancements during active listening to ecologically valid continuous speech (Forte et al., 2017; Etard et al., 2019; Saiz-Alía et al., 2019).

A larger body of FFR literature supports the supposition that short- and long-term training induce neuroplastic changes in the auditory subcortex over time. Studies in which listeners were trained to discriminate stimuli by focusing on a specific sound feature (e.g., global pitch or dynamic pitch contours) have reported enhancement of the neural representation of the trained feature (e.g., Russo et al., 2005; Song et al., 2008, 2012; Carcagno and Plack, 2011; Chandrasekaran et al., 2012; Skoe et al., 2014). These changes were noted after multiple hours or days of training; however, additional studies have reported rapid FFR modulation occurring within minutes of training onset (e.g., Skoe and Kraus, 2010; Skoe et al., 2013). Similar and more robust enhancements are observed in musicians (Wong et al., 2007; Bidelman and Krishnan, 2009) and tonal language speakers (Krishnan et al., 2005; Swaminathan et al., 2008; Krishnan and Gandour, 2009) who, by virtue of their lived experiences, have undergone a form of long-term auditory training (see Kraus and Chandrasekaran, 2010; Strait and Kraus, 2014; Kraus and White-Schwoch, 2015 for reviews).

The majority of FFR studies examining online or training-related changes in neural function have focused on neural representation of the speech envelope and its harmonics (FFR_*ENV*_). Recent evidence suggests that although the FFR_*ENV*_ arises primarily from the auditory subcortex (Chandrasekaran and Kraus, 2010; Bidelman, 2015; Bidelman and Powers, 2018; Bidelman et al., 2018a), cortical contributions may also be present, particularly for stimuli with fundamental frequencies < ∼150 Hz (Coffey et al., 2016, 2019). This new understanding of FFR_*ENV*_ origins presents the possibility that neuroplastic changes observed in some previous studies may not be constrained to the subcortex. One way to ensure that measured neural responses are biased exclusively toward subcortical generators is to use stimuli comprised of behaviorally-significant higher frequency (>200 Hz) speech content, as more caudal generators begin to dominate the FFR with increasing stimulus frequencies (Gardi et al., 1979; Galbraith et al., 2001; Tichko and Skoe, 2017).

Sine-wave speech (SWS) is an acoustically manipulated form of speech in which formant trajectories are represented by time-variant sine waves, and the remainder of the acoustic signal is discarded (Remez et al., 1981). It can therefore be conceptualized as speech “fine structure” that has been spectrally reduced to two or three dynamic frequency components. The range of average first (F1) and second (F2) formant frequencies in adult American English speakers is ∼300–775 and ∼900–2,700 Hz, respectively (Lindblom, 1990). Because the upper frequency limit of the FFR is approximately ∼1,200–1,300 Hz (Bidelman and Powers, 2018), much of the F1 and F2 formant space may be captured by FFRs evoked by SWS (FFR_SWS_). A critical advantage of SWS is that naïve listeners do not hear it as speech (Remez et al., 1981; Barker and Cooke, 1999; Möttönen et al., 2006); however, with minimal instruction and/or training, listeners achieve a high level of SWS comprehension. Consequently, it is possible to use identical speech-like stimuli to evoke FFR_SWS_ pre- and post-engagement of the auditory efferent system through online or brief short-term training activities. While the neural networks involved in this top-down process are not fully understood, recent reports examining cortical responses to SWS (or vocoded speech) indicate that activity from different brain networks is involved based on whether the signals are perceived as speech or non-speech (Davis and Johnsrude, 2003; Eisner et al., 2010; Hervais-Adelman et al., 2012; Khoshkhoo et al., 2018). Specifically, SWS is represented in the auditory cortex based on “bottom-up” acoustic features in naïve listeners. When listeners undergo a perceptual shift and begin to understand these degraded signals as speech, left inferior frontal cortex activity increases significantly while auditory cortex activity remains stable (Khoshkhoo et al., 2018). Given the observations that active listening sequentially modulates neural tuning in the same cortical networks in a top-down fashion (e.g., Atiani et al., 2014), it is possible that these modulatory effects continue into the auditory brainstem *via* the efferent system (Bidelman et al., 2019; Price and Bidelman, 2021).

In the present experiment, three SWS tokens, differing mainly in their F1 contours, were used to evoke FFR_SWS_ before and after a brief auditory training paradigm in which listeners were informed that they were listening to degraded speech and were asked to classify each token. These results were compared to FFR_SWS_ measured from a control group, which did not undergo training. FFR_SWS_ were confirmed to be of neural origin with latencies suggesting brainstem generators and high stimulus-to-response cross-correlations. In the test group, all SWS stimuli were rapidly perceived as speech when presented with a SWS carrier phrase in a brief training phase, and average token identification reached ceiling performance within 25 training trials or less per stimulus. FFR_SWS_ amplitudes in the test group were enhanced post-training for each stimulus compared to the control group. Further, linear support vector machine classification of FFR_SWS_ significantly improved post-training in the test group compared to controls, indicating that training-induced neural enhancements were sufficient to bolster machine learning classification accuracy. These results suggest that the efferent auditory system may rapidly modulate auditory brainstem representation of sounds depending on their context and perception as non-speech or speech.

## Materials and Methods

### Participants

This study was approved by the University of Texas at Austin Institutional Review Board. Eighteen adults (mean age = 22.2 years) with no history of audiologic or neurologic injury were enrolled. Half of the participants were placed in a training group and the other half served as untrained controls. Participants had normal hearing (≤25 dB HL) from 250 to 8,000 Hz bilaterally. Each participant provided written consent and completed 3 h of testing for which they were compensated.

### Sine Wave Speech Stimuli

**FIGURE 1:**
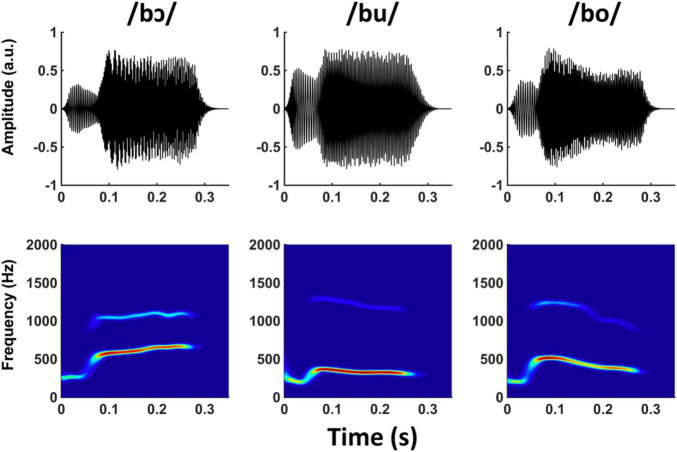
Waveforms and spectrograms of /bɔ/, /bu/, and /bo/ SWS stimuli.

Three naturally produced CV speech tokens, /bɔ/, /bu/, and /bo/, were recorded (44,100 Hz sampling rate) from an adult male speaker with a Standard American English accent. The speaker was told to maintain constant voice pitch across all recordings. Each CV token was 335 ms in duration, and cosine squared ramps were applied to the last 50 ms of each stimulus to equate and smooth offsets across stimuli. The natural CV tokens were then converted to SWS in Praat software (Boersma, 2009) using the approach developed by Darwin (2003). This approach uses linear predictive coding analysis to identify formant center frequencies and amplitudes within a sliding window over the stimulus. The formants are then replaced with time-varying sinusoids, and all other speech content is discarded (Figure 1). Only the first two formants from the original stimuli were kept, as FFRs were unlikely to be evoked by higher frequency formants. A carrier phrase (“The word is ____.”) that was only used in the brief training phase for the training group (described below) was also converted to SWS in the same manner described above. All SWS stimuli were RMS normalized to ensure equal presentation level.

The three vowels in the CV stimuli were selected for multiple reasons related to their relative positions in the F1/F2 formant space. First, phase-locking in the auditory nervous system becomes poorer as stimulus frequency increases. Consequently, stimuli comprised of lower frequencies generate more robust FFR responses (e.g., Bidelman and Powers, 2018). The vowels /ɔ/, /u/, and /o/ have the lowest possible F1 and F2 frequencies in American English and are therefore the most ideal SWS candidates for evoking robust FFRs_*WS*_. Second, the vowels primarily differ in their F1 contours, whereas the F2 contours are less disparate. The range in F1 frequencies for the three vowels was ∼300–675 Hz, whereas the range in F2 was ∼1,100–1,300 Hz. Because CV differences were most pronounced in their F1 frequencies, we anticipated that listeners in the training group would primarily focus on this feature to successfully complete the auditory training task and that neural enhancement related to the brief training period would be apparent at the F1 frequency (described below). Third, the total range of F1 and F2 stimulus frequencies (∼300–1,300 Hz) biases the FFR to reflect more caudal subcortical generators (e.g., Galbraith et al., 2001; Bidelman, 2018).

### Procedure

#### Training Group

All experimental procedures occurred in a double-walled sound booth with participants seated in a reclining chair. Auditory stimuli were presented diotically through electromagnetically shielded ER-3 insert earphones (Etymotic Research, Elk Grove Village, IL), and visual prompts (used only in the training phase) were presented through a Dell PC monitor. Experiment stimuli were programmed and controlled *via* Neuroscan’s GenTask module (Compumedics Neuroscan, Charlotte, NC). The experiment began with a *pre-training phase* in which FFR_SWS_ were evoked by /bɔ/, /bu/, and /bo/ SWS tokens presented in random order. Half of the stimulus presentations were in one polarity (“Polarity A”) and half were in the opposite polarity (“Polarity B”). Each stimulus was presented in each polarity 1,000 times for a total of 6,000 sweeps. The intertrial interval between stimuli was 600 ms. During the pre-training phase, each participant was asked to remain still while quietly watching a subtitled movie or show of his or her choosing.

The *training phase* of the experiment began upon conclusion of the pre-training phase and after a brief break. Participants were notified that the stimuli they were hearing in the previous block were modified speech signals and that the training phase would require them to learn and identify the speech signals using a response keypad (Compumedics Neuroscan, Charlotte, NC). No additional instruction was given. At the beginning of each training trial, participants heard the SWS carrier phrase *“The word is _____.,”* with one of the three SWS tokens randomly presented as the target word. Simultaneously to the auditory presentation of the carrier phrase and target word, the participants saw a visual prompt on a monitor located directly in front of them and outside of the sound booth. The prompt depicted a visual representation of the carrier phrase with a blank in the target word space, exactly as written in the italicized quote above. The total duration of the carrier phrase and target word was 1,500 ms; an additional 500 ms of silence was appended to the end of each carrier and target presentation to encourage participants to remain still prior to pressing the response keypad to submit their answer following the next prompt. Participants then saw a slide on the monitor with possible target words written non-phonetically as “bah,” “boo,” or “bow.” The participant indicated which SWS word was heard by pressing one of three buttons on the response keypad, which was then followed by a 600 ms intertrial interval. Visual feedback (“Correct” or “Incorrect”) was then given to participants, which was followed by another 600 ms interval before the onset of the next trial. This procedure was repeated 25 times per stimulus for a total of 75 training trials. Relatively few training trials were chosen based on previous reports that SWS becomes rapidly intelligible with very little training (Remez et al., 1981; Möttönen et al., 2006).

A *testing phase* followed the training phase. The main purpose of the testing phase was to ensure that participants retained SWS identification accuracy in the absence of the carrier phrase, which provided additional “samples” of the speaker’s formant structure. In the testing phase, each trial began with the random presentation of a SWS target token. After a 600 ms pause, participants were invited to indicate their responses on a keypad, using the same slide described above with written target words as a reference. Participants had 900 ms from the onset to indicate their responses. Feedback was not provided in the test phase. Following training and testing phases, a *post-training phase*, parametrically identical to the pre-training phase, was conducted.

#### Control Group

The control group underwent passive FFR_SWS_ measurements that were identical to pre-training and post-training measurements in the training group. In place of the SWS training and testing phases, the control group was asked to watch an unrelated captioned television show and answer comprehension questions related to its content. While control group participants watched the captioned television show, they were exposed to the same carrier sentences as the test-group; however, they were never told that they were hearing modified speech at any point of the experiment. None of the control participants perceived the SWS stimuli to be speech according to a post-experiment survey. The purpose of including the control group in this study was to determine if pre- and post-training FFR_SWS_ enhancements in the test group were simply related to *exposure* to the SWS stimuli during the recording session and not due to efferent modulation following a perceptual shift from non-speech to speech perception. Note that, for simplicity, we refer to the first and second passive FFR_SWS_ measurements for test and control groups as “pre- and post-training” measurements throughout the manuscript, even though the control group did not undergo auditory training.

### EEG Acquisition and Pre-processing

Electrophysiologic responses were obtained with a Neuroscan SynAmps2 system (Compumedics Neuroscan, Charlotte, NC). Responses were recorded at a 5,000 Hz sampling rate *via* a single-channel bipolar montage, Fpz (+), C7 vertebra (−), forehead (GND), and amplified by a factor of 100,000. Continuous data were exported from Curry 8 software, and further analyses were performed offline in MATLAB (The MathWorks, Natick, MA). All continuous data were first bandpass filtered from 100 to 2,400 Hz. For pre- and post-training FFR_SWS_, continuous responses were epoched from −50 to 550 ms (re: SWS token onset), and single-trial responses were grouped by stimulus type. Responses were corrected for insert earphone delays by subtracting 1 ms from the epoched data. Epochs were detrended, artifact rejected at ± 50 μV, and baseline corrected. Remaining sweeps were used to create grand average FFR_SWS_ for each stimulus such that individual polarities (A and B) as well as “added” [(A+B)/2] and subtracted [(A−B)/2] waveforms could be independently evaluated. Individual polarities and subtracted waveforms were used in a cross-correlation analysis (described below) to verify that FFR_SWS_ were neural in origin. Added polarity responses are generally used to accentuate neural representation of the envelope (Aiken and Picton, 2008). Because the stimuli in the present study did not have envelopes, added polarity waveforms were evaluated mainly as a quality control measure to ensure that FFR_SWS_ were not obliterated (which indicates that the measured responses are stimulus artifact or cochlear microphonic). In some cases, low amplitude waveforms containing energy at F1*2 were observed in the added polarity. This likely occurs because phase locked neural responses evoked by one stimulus polarity are temporally shifted by a half-cycle relative to the opposite polarity due to half-wave rectification (see Aiken and Picton, 2008; Lichtenhan et al., 2013). Adding these responses together can produce a doubling of the stimulus frequency and provides additional evidence that the measured responses are from *neural* generators.

## Analyses

### Test Group Training- and Testing-Phase Response Accuracy and Reaction Time

Training group response accuracy and reaction time were evaluated using behavioral data from training and test phases, respectively, as both measures are indicative of auditory training effects (e.g., Ritter et al., 1972; Song et al., 2008). Response accuracy, defined binarily on each trial as “correct” or “incorrect,” was analyzed using mixed effects logistic regression with trial number and stimulus type as independent variables. Reaction time, defined as the post-stimulus onset time (re: to SWS target) at which respondents pressed the response keypad to indicate their choice, was evaluated using multiple linear regression with trial number and stimulus type as independent variables.

### Stimulus-to-Response Cross-Correlation

Electromagnetic stimulus artifact, cochlear microphonic, and FFR waveforms can all mimic periodic characteristics of the input stimulus. A common method used to evaluate whether measured electrophysiologic responses are from neural generators or non-neural contaminants is to perform a cross-correlation between the stimulus and response. In this procedure, correlations between stimulus and FFR waveforms are calculated as the FFR waveform is temporally shifted relative to the stimulus waveform on a point-by-point basis (Skoe and Kraus, 2010). The time lag that produces the largest correlation coefficient is an estimated delay between stimulus and response. Responses generated by the auditory nerve and brainstem are expected to have a delay of ∼3–10 ms, depending on the electrode montage, stimulus frequency, and interaction between multiple neural generators as they reach scalp electrodes (e.g., Galbraith et al., 2001; Tichko and Skoe, 2017; Bidelman, 2018). In contrast, cochlear microphonic (arising from hair cell alternating currents primarily in the basal tail of the basilar membrane traveling wave; see Eggermont, 2017 for review) and stimulus artifact have short delays of ∼0–1 ms (Gardi et al., 1979). Stimulus-to-response cross-correlations were calculated for individual polarities (A and B) and subtracted waveforms evoked by each SWS stimulus in the pre- and post-training phases for test and control groups. SWS stimuli were first down-sampled from 44,100 to 5,000 Hz to match the FFR_SWS_ sampling rate, resulting in 0.2 ms precision in delay estimates. The maximum possible time delay producing the largest correlation coefficient was constrained between ±20 ms. Responses for which the estimated delays were within 3–10 ms were considered to be of neural origin. These responses were kept for further analysis. Cross-correlation coefficients, which are constrained between −1 and 1 and are non-normally distributed, were transformed to Fisher *z*-values (Cohen et al., 2013). A three-way multiple analysis of variance (MANOVA) with repeated measures was conducted to assess the impacts of group (test vs. control). training status (pre- vs. post-training), and stimuli (/bɔ/, /bu/, and /bo/) on participants’ FFR_SWS_ latencies and z-transformed cross-correlation coefficients.

### Fourier Analyzer

In contrast to a Fourier transform, which is commonly used to analyze steady-state stimuli/responses, a Fourier analyzer (FA) provides a better estimate of response amplitudes at frequencies of interest for signals with time-varying spectra (Aiken and Picton, 2006). Because FFRs are expected to follow dynamic frequency changes of a stimulus over time, the FA uses the stimulus frequency trajectory as a “reference” to detect FFR spectral amplitudes at frequencies along this trajectory by integration (Aiken and Picton, 2006). The stimuli used in the present study have non-stationary F1 and F2. Therefore, an FA was implemented to calculate response amplitudes in frequency bins corresponding to F1 and F2 trajectories to determine the strength of neural phase locking to each simulated formant.

We used a similar approach to implement FA as described by Aiken and Picton (2006) and Choi et al. (2013). First, stimulus reference tracks following F1 and F2, respectively, were created by exporting only F1 or F2 SWS sine-waves from Praat. Complex representations of F1 and F2 stimuli were obtained by Hilbert transform, and the instantaneous phase was calculated by finding the angle of the output of the Hilbert transform. F1 and F2 instantaneous frequencies were then calculated as the derivatives of the unwrapped phases at each time point. Since calculating the derivatives in this manner is equivalent to applying a high-pass filter, it introduces sharp perturbations in the resulting frequency tracks. Consequently, we smoothed the obtained instantaneous frequencies across time by applying a 50-point boxcar moving average 3 times. Reference complex sinusoids were then created for F1 and F2 frequency tracks using the instantaneous phase angles for each stimulus.

As mentioned above, FFRs demonstrate a characteristic delay between stimulus and response of ∼3–10 ms due to neural conduction time. FFR_SWS_ waveforms were shifted by – 6 ms based on pilot data testing to correct for neural delays and ensure that reference tracks were, on average, temporally aligned with the FFR_SWS_ waveforms prior to integration (Purcell et al., 2004; Aiken and Picton, 2006). Reference tracks and FFR_SWS_ waveforms were then integrated by multiplying the two waveforms over time (Choi et al., 2013) and computing the mean of the obtained complex numbers. The absolute value and the angle of the mean were then calculated as the FFR amplitude and the phase over the duration of the response (50–335 ms), respectively.

In order to determine whether FFR_SWS_ amplitudes at F1 and F2 were above background noise levels, 10 adjacent frequency tracks were also created to measure response amplitudes at non-stimulus frequencies. Five noise tracks above and five below each F1 and F2 track were obtained by adding or subtracting a fixed number of cycles per second. Noise tracks began at F1 ± 5 and F2 ± 5 Hz, respectively, and increased or decreased in 1 Hz steps. The same FA procedures as above were then used to estimate noise levels in the 10 adjacent non-stimulus frequency bins. F1 and F2 responses were deemed “present” if their amplitudes exceeded the noise floor averaged across 10 adjacent frequency bins. This approach is more lenient than other statistically based methods for determining response presence/absence (e.g., *F*-tests or Hotelling’s *T*^2^-tests; see Picton et al., 2003 for review). However, because the primary focus of this study was to evaluate potential *enhancement* of FFR_SWS_ following perceptual shifts, we did not want to remove participants who had low baseline FFR_*SWS.*_

### Machine Learning Classification of FFR_SWS_

Previous experiments have used machine learning algorithms to assess whether the information contained in FFRs is sufficient to decode the stimulus classes that evoked them (Sadeghian et al., 2015; Holdgraf et al., 2017; Llanos et al., 2017; Yi et al., 2017; Xie et al., 2018, 2019). Under this approach, FFR classification performance (i.e., the accuracy with which FFRs are correctly classified by the machine learning algorithm) serves as an objective measure of stimulus discrimination. Importantly, FFR classification accuracy can be compared between levels of an independent variable (e.g., training or attention conditions) to determine how these factors impact classification performance (e.g., Xie et al., 2018). The rationale is that, if attention or training modulate neural function as captured by the FFR, the accuracies with which FFRs are classified should reflect this modulation *via* improving (enhancement) or declining (suppression) classification accuracy.

A MATLAB-constructed linear support vector machine (SVM; Cristianini and Shawe-Taylor, 2000) was used to classify pre- and post-training FFR_SWS_ for test and control groups following the general procedures described by Xie et al. (2019). We first epoched all subtracted FFR_SWS_ waveforms from 0 to 380 ms and used these 1,900 amplitude-by-time points as linear SVM input features. The model outputs were stimulus type (/bɔ/, /bu/, and /bo/). Because standard linear SVM can only classify data into binary classes, a one-against-one strategy was used. In this approach, the linear SVM constructs N(N-1)/2 classifiers, where N is the number of classes; *N* = 3 in this experiment, as three SWS stimuli were used. After FFR classification is performed on all possible pairwise combinations, the class with the highest accuracy is used as the classification label.

The model was cross-validated using a three-fold approach that was repeated 2,500 times (see Xie et al., 2019; Figure 1). For each iteration of the linear SVM classifier, participants were randomly and equally divided into 3 groups (or folds). A “leave-one-out” strategy then used two of the three-folds to train the classifier. After training the classifier, the held-out fold was used as test data. This was repeated within each iteration such that each fold was held-out as the test data and the other two-folds were used for training the classifier. The average classifier accuracy across cross-validations was calculated for each iteration. Outcomes of the 2,500 iterations were also used to create grand total cross-validation accuracies as well as a distribution of accuracies. A null distribution of model accuracies was also generated using the steps above, with the exception that model outputs (i.e., stimulus labels) were randomly assigned to FFR_SWS_ inputs on each iteration of the loop. Statistical significance of “true” classifier performance was determined using *p* = (*a* + 1)/(*n* + 1), where *a* denotes the number of observations from the null classification distribution that surpasses the median of the “true” distribution and *n* is the total number of observations comprising the null distribution (Phipson and Smyth, 2010, as cited in Xie et al., 2019). The same equation was also used to test whether pre- and post-training FFR_SWS_ classification accuracy distributions were significantly different for test and control groups.

## Results

### Test Group Training- and Testing-Phase Behavioral Performance

**FIGURE 2:**
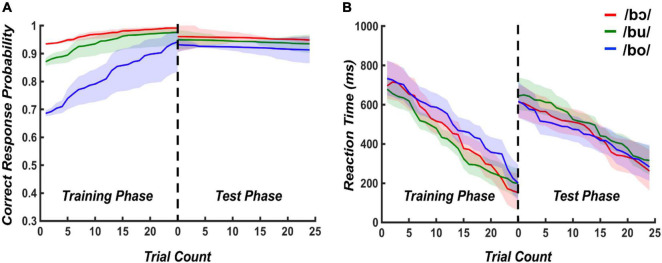
Modeled accuracy and reaction times for training and testing phases. Logistic and multiple linear regression model outputs using stimulus type and trial count as predictors were used to plot predicted accuracy **(A)** and reaction times **(B)**, respectively. Shading indicates 95% confidence intervals of each modeled response.

Modeled accuracy and reaction times for training and test phases are plotted in Figure 2. For the training phase, the mixed effects logistic regression model containing training time and stimulus type as predictors was statistically significant [*X^2^(2)* = 37.31, *p* < 0.001]. When holding stimulus type constant, the odds of a correct response increased by 3% [95% CI (0.13, 0.47)] for a one-unit increase in trials. When holding trial count constant, the accuracy decreased by 6% [95% CI (−0.76, −0.41)] when changing from /bɔ/ to /bu/ and /bu/ to /bo/. The multiple linear regression model evaluating reaction time suggested that training time and stimuli explained 9% of the variance, [*R*^2^ = 0.09, *F*_(2, 672)_ = 33,28, *p* < 0.001]. When holding stimulus type constant, training time significantly predicted reaction time, β = −7.73, *t* = −8.09, *p* < 0.001, suggesting the reaction time decreased when the training time increased. When holding training time constant, stimulus type did not significantly predict reaction time (β = 15.62, *t* = 0.62, *p* = 0.54).

For the test phase, the mixed effects logistic regression model containing test time and stimuli as predictors was not statistically significant [*X^2^(2)* = 5.38, *p* = 0.07]. The results of multiple linear regression revealed that testing time and stimuli explained 3% of the variance, [*R*^2^ = 0.03, *F*_(2, 672)_ = 12,8, *p* < 0.001]. When stimulus type was held constant, testing time significantly predicted reaction time, β = −4.98, *t* = −5.06, *p* < 0.001. However, there is no significant prediction of stimuli on reaction time (β = 12.66, *t* = 0.95, *p* = 0.34). These results collectively suggest that accuracy improved with more exposure to the stimuli in the training phase, with /bɔ/ and /bu/ being more rapidly attained than /bo/. Further, all stimuli were discriminated with a high level of accuracy during the test phase. Irrespective of the target stimulus, reaction time similarly decreased during training and testing phases.

### Stimulus-to-Response Cross-Correlations

Initial stimulus-to-response calculations for pre- and post-training FFR_SWS_ showed sharply peaked cross-correlation functions between /bu/ and /bo/ and their respective SWS stimulus waveforms; because the stimulus waveforms are dominated by the F1 component, these results indicated strong neural phase locking to F1. In contrast, cross-correlations for the /bɔ/ were poor despite these FFR_SWS_ waveforms being highly periodic. Spectrographic analysis of the average FFR_SWS_ to /bɔ/ demonstrated that the neural response was in fact phase-locked to the quadratic distortion product (F2–F1) instead of F1. The F2–F1 distortion product is mechanically initiated by interactions between F2 and F1 traveling waves on the basilar membrane, and the nervous system can phase lock to this and other distortions as it would to acoustically-delivered stimuli of the same frequency (e.g., Siegel et al., 1982; Smith et al., 2017). Because F2–F1 is not present in the acoustic stimulus, the neural response does not bear a resemblance to the stimulus. To determine whether F2–F1 frequency tracking for /bɔ/ SWS_*FFR*_ were of neural origin, we approximated an F2–F1 “stimulus” waveform by taking the analytic envelope of the original SWS stimulus and band-passing it between 100 and 2,400 Hz. Cross-correlations were then rerun between the F2–F1 stimulus waveform and /bɔ/ SWS_*FFR.*_ With this adjustment, /bɔ/ SWS_*FFR*_ cross-correlation functions demonstrated sharp peaks similar to the other responses.

**FIGURE 3:**
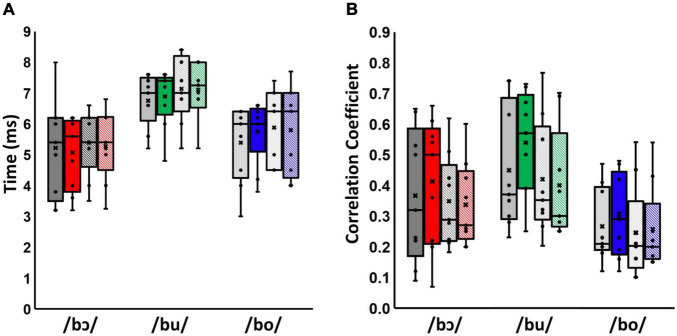
Latency **(A)** and cross-correlation strength **(B)** for /bɔ/, /bu/, and /bo/ FFR_SWS_. Pre-training responses are shown in gray and post-training responses are shown in color. Test group responses are solid-filled bars, whereas control group responses are cross-hatched. Means and medians are denoted by Xs and horizontal lines, respectively. Note that latencies and cross-correlations for /bɔ/ were calculated using the F2–F1 waveform as the “stimulus,” as described in the text.

Results of the cross-correlation analyses for test and control groups are shown in Figure 3. Multivariate analysis showed a significant effect of stimulus type on both latency and cross-correlation strength across groups and training status, [Wilks’ Lamda = 0.33, *F*_(4, 62)_ = 11.42, *p* < 0.001, η^2^ = 0.42], suggesting that stimulus type affected cross-correlation strength and latencies of neural responses. The effect size, calculated using eta squared, indicated that this stimulus type effect accounted for 42% of the variance in cross-correlation strength and latency. Moreover, there was a significant effect of training status between test and control groups across stimuli, [Wilks’ Lamda = 0.66, *F*_(2, 15)_ = 3.82, *p* < 0.05, η^2^ = 0.34], suggesting that the interaction of training status and group affected the cross-correlation strength and latency of neural responses. The effect size, calculated using eta squared, indicated the interaction of training status and groups effect accounted for 34% of the variance in cross-correlation strength and latency. However, there is no significant stimuli*group [*F*_(4, 62)_ = 0.12, *p* = 0.98, η^2^ = 0.01], training status [*F*_(2, 15)_ = 1.94, *p* = 0.18, η^2^ = 0.21], stimuli*training [*F*_(4, 62)_ = 0.59, *p* = 0.67, η^2^ = 0.03], stimuli*training*group [*F*_(4, 62)_ = 0.12, *p* = 0.98, η^2^ = 0.01] effect on cross-correlation strength and latency [*F*_(2, 7)_, *p* = 0.49, η^2^ = 0.19].

Univariate tests were used to further examine the effects on latency and cross-correlation strength. These results show a significant stimulus effect on latency [Greenhouse-Geisser = 57.72, *F*_(1.88, 30.08)_ = 15.93, *p* < 0.001, η^2^ = 0.50] and cross-correlation strength [Greenhouse-Geisser = 0.62, *F*_(1.99, 31.87)_ = 11, *p* < 0.001, η^2^ = 0.41]. Moreover, there was a significant effect of the interaction between training status and groups on cross-correlation strength [Greenhouse-Geisser = 0.02, *F*_(1, 1.68)_ = 4.62, *p* < 0.05, η^2^ = 0.22]. Within-subjects contrasts showed that latency in the /bu/ condition was significantly higher than /bɔ/ [*F*_(1, 16)_ = 39.64, *p* < 0.001, η^2^ = 0.71] and /bo/ conditions [*F*_(1, 16)_ = 14.59, *p* < 0.01, η^2^ = 0.48]. Additionally, cross-correlation strength in /bu/ was higher than /bo/ [*F*_(1, 16)_ = 23.29, *p* < 0.001, η^2^ = 0.59]. Lastly, there was a significantly higher cross-correlation after training than before training in test group [*F*_(1, 16)_ = 4.61, *p* < 0.05, η^2^ = 0.22]; this significant difference is larger than the pre- and post-training difference in control group.

### Pre- and Post-training FFR_SWS_ Fourier Analyzers

FFR_SWS_ amplitudes at F1 and F2 were calculated over the duration of the response using FAs. These calculations produced a single amplitude estimate representing the strength of neural phase locking to the stimulus feature of interest over the entire duration of the stimulus. FFR_SWS_ to /bu/ and /bo/ produced measurable F1 responses above the noise floor for all subjects in pre- and post-training measurements. The issue described above regarding neural phase locking to F2–F1 in /bɔ/ FFR_SWS_ also impacted our initial FA calculations for the /bɔ/ stimulus such that F1 was not robustly represented. Consequently, we used the F2–F1 “stimulus” waveform to create an F2–F1 frequency track for /bɔ/ responses, using identical procedures described in the method section. Using this approach yielded measurable F2–F1 neural responses in every participant for /bɔ/ in pre- and post-training measurements. The following analyses focus on F1 amplitudes for /bu/ and /bo/ and F2–F1 amplitudes for /bɔ/. Because F2 was only measurable in <25% of responses, we did not further analyze these components.

**FIGURE 4:**
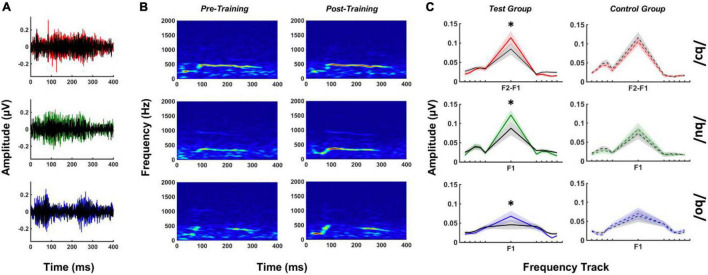
FFR_SWS_ pre- and post-training waveforms **(A)**, spectrograms **(B)**, and FA results **(C)** for each stimulus. **(A,B)** Are from test group data only, whereas **(C)** includes test and control FA results for comparison. Colored (red, green, and blue) waveforms and FA spectra represent post-training responses, whereas black waveforms are their pre-training counterparts. FA center frequencies (F2–F1 or F1) are denoted for each FA plot; noise bins starting at ± 5 Hz relative to the frequency of interest are indicated by peripheral tick marks on the *x*-axis (shading = SEM). All results represented subtracted waveforms. *p < 0.05.

Figure 4 depicts mean FFR_SWS_ waveforms and spectrograms for pre- and post-training test group responses as well as test and control group FA results for each stimulus. All post-training FFR_SWS_ responses are larger in amplitude than pre-training responses for the test group, which can be seen in waveform (Figure 4A) and spectrographic (Figure 4B) representations. Examination of the FA results for the test group (Figure 4C) demonstrates that these enhancements are at the frequency of interest only and are not observed in the adjacent noise bins. By comparison, FFR_SWS_ enhancements are not apparent in the control group FA responses.

The effect of training status and group on FA amplitudes was analyzed using a two-way MANOVA with repeated measures. This analysis showed an interaction of training status and group [*F*_(5, 12)_ = 3.09, *p* = 0.05, η^2^ = 0.56] on FA amplitude. Univariate tests revealed significant interactions between training status and group for /bɔ/ [Greenhouse-Geisser = 0.002, *F*_(1, 16)_ = 6.72, *p* < 0.05, η^2^ = 0.30], /bu/ [Greenhouse-Geisser = 0.004, *F*_(1, 16)_ = 8.44, *p* < 0.05, η^2^ = 0.34], and /bo/ [Greenhouse-Geisser = 0.002, *F*_(1, 16)_ = 9.96, *p* < 0.01, η^2^ = 0.38] FA amplitudes. Pre- and post-training differences between test and control groups revealed that /bɔ/ [*F*_(1, 16)_ = 6.73, *p* < 0.05, η^2^ = 0.30], /bu/ [*F*_(1, 16)_ = 8.44, *p* < 0.05, η^2^ = 0.35], and /bo/ [*F*_(1, 16)_ = 9.96, *p* < 0.01, η^2^ = 0.38] FA amplitudes were significantly higher after training in test group but not in the control group.

### Machine Learning Classification of FFR_SWS_

**FIGURE 5:**
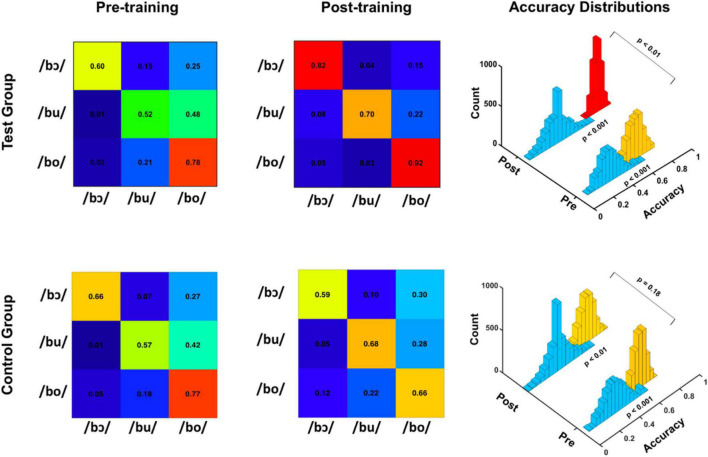
Pre-training and post-training classification accuracies for test (top) and control (bottom) groups. Confusion matrices on the left demonstrate linear SVM classification accuracies for pre-training FFR_SWS_, whereas the confusion matrices in the center demonstrate post-training accuracies. 3D histograms on the right show empirical null distributions (teal bins) and pre- and post-training average classification accuracy distributions for test and control groups. Pre- and post-training distributions were significantly above the null distribution for test and control groups. Additionally, the test group post-training distribution was significantly more accurate than the pre-training distribution.

Average linear SVM classification accuracies for test and control groups are depicted for pre-training and post-training FFR_SWS_ in the confusion matrices of Figure 5. For the test group, pre-training classification accuracy was poorer for each stimulus classifier relative to post-training accuracy, whereas control group pre- and post-training classification results do not follow a clear pattern. Overall classification accuracy distributions representing all 2,500 iterations are depicted in the 3D histogram plots, as are empirical null distributions generated by randomly shuffling classifier outputs (i.e., response labels) for each iteration. Pre-training (*p* < 0.001) and post-training (*p* < 0.001) FFR_SWS_ classification was significantly above the null distribution for test and control groups, as determined using *p* = (*a* + 1)/(*n* + 1). In the test group, post-training classification was significantly higher (*p* < 0.01) than pre-training classification using the same equation. In contrast, pre- and post-training classification were not different in the control group (*p* = 0.18). Collectively, these results indicate that classification of pre- and post-training FFR_SWS_ was significantly above chance for test and control groups; however, post-training data were classified with significantly higher accuracy than pre-training data in the test group compared to the control group.

## Discussion

To our knowledge, this is the first study using FFR_SWS_ to examine training or context effects in the auditory brainstem. Utilizing SWS in this context allowed for direct comparisons between pre- and post-training FFR_SWS_ evoked by identical, acoustically-sparse speech stimuli that initiated neural responses from more caudal subcortical sources than stimuli used in previous reports. Because most listeners do not hear SWS as speech unless they are provided with additional instruction (Remez et al., 1981; Möttönen et al., 2006), pre-training neural representation of SWS theoretically offers a glimpse into bottom-up auditory processing of the “naïve” auditory nervous system. When additional instruction or context is provided to listeners regarding SWS, they often attain a high level of comprehension in a brief period of time or, in some cases, immediately (Remez et al., 1981; Möttönen et al., 2006). Thus, post-training FFR_SWS_ may offer insight into how rapidly and potently the auditory brainstem can be functionally modulated *via* the efferent system when speech comprehension networks are engaged.

Our behavioral results suggest that SWS stimuli were rapidly attained in the training trials, albeit at slightly different rates. For example, /bɔ/ and /bu/ discrimination reached peak performance within relatively few trials, whereas /bo/ required more training before responses were consistently accurate. This pattern suggests that /bo/ was initially more difficult to discriminate than /bɔ/ and /bu/, which may simply be explained by acoustical differences (i.e., /bo/ and /bu/ F1 and F2 contours are more similar than /bɔ/ and were spaced such that they were unlikely to generate strong distortion products; see Figure 1). An additional revelation from our FFR_SWS_ data was that participants may have benefitted from hearing the F2–F1 distortion product created by the /bɔ/ stimulus. The F2–F1 frequency is generated by mechanical interaction on the basilar membrane and “feeds forward” into the auditory nervous system, as do other distortion products (Siegel et al., 1982; Chertoff et al., 1992; Dhar et al., 2009; Smith et al., 2017), effectively converting a dynamic two-tone stimulus into a perceptually richer input (Goldstein et al., 1978). Because we did not assess psychophysical weighting of the F2–F1 cue, it is not clear whether its post-training enhancement in the FFR was a consequence of direct attention to this cue or a gross upscaling of any auditory stimuli relevant to the perceptual task.

Despite stimulus-related differences in behavioral training results, we observed that all FFR_SWS_ in the test group were enhanced in the post-training phase relative to the pre-training phase and compared to a control group. This was indicated in larger FA amplitudes of F1 (for /bo/ and /bu/) and F2–F1 (for /bɔ/), as well as higher machine learning classification accuracy in the post-training phase. Importantly, these differences were due to FFR_SWS_ amplitude enhancement and not differences in residual noise between pre- and post-training responses, as evidenced by the FA noise tracks (Figure 4). There are multiple potential explanations for the observed FFR_SWS_ enhancements in the test group. First, the rapid perceptual shift from non-speech to speech may have engaged speech comprehension networks originating in frontal cortex and extending through auditory cortex and brain stem (Davis and Johnsrude, 2003; Eisner et al., 2010; Hervais-Adelman et al., 2012; Bidelman et al., 2018a,^2019^; Khoshkhoo et al., 2018). That FFR_SWS_ enhancements reflect a more immediate *online* context shift and not short-term training *per se* is supported by a few congruent observations in our behavioral and neurophysiologic data. For example, perceptual shifts appear to have occurred quickly for /bɔ/ and /bu/ stimuli, whereas /bo/ required more exposure trials before it was attained. These results suggest that perceptual salience of the context shift may have differed slightly across stimuli. The size of FA enhancements and within-class changes in linear SVM classification accuracy between pre- and post-training trials mirror the behavioral results: more immediate behavioral SWS identification was associated with larger FA enhancements and greater changes in classification accuracy post-training. It is also notable that the enhancements observed in the present study appeared earlier than many reports on FFR training effects, which required multiple hours to days of training (e.g., Song et al., 2008; Carcagno and Plack, 2011). This may be related to the fact that SWS was initially processed as a completely different class of stimulus (e.g., uncorrelated “whistles”) before being recognized as speech, whereas participants in previous studies were aware from experiment onset that they were hearing speech or music stimuli. Further, SWS forces listeners to focus on a minimal number of cues (F1, F2, and/or F2–F1), whereas speech and music pitch may be determined in a variety of ways, such as listening to resolved and/or unresolved harmonics (e.g., Laudanski et al., 2014); therefore, attention may be allocated to different channels of information summating to produce the FFR. A limitation of our approach, which does not allow us to resolve single-trial FFR_SWS_, is that we cannot delineate whether the observed enhancements are related to online or short-term changes following the perceptual shift.

A control group was used in the present study to examine whether post-training vs. pre-training differences were simply a result of more exposure to the stimuli during the experimental protocol. Our results suggest that this is not the case, as the control group responses were not enhanced “post-training” relative to “pre-training.” These results comport with the multiple studies that have demonstrated high test-retest reliability of FFR amplitudes within and between *passive* test sessions (e.g., Song et al., 2011; Bidelman et al., 2018b; Easwar et al., 2020).

Future studies will examine afferent-efferent connectivity using similar SWS stimuli to examine the time course and neural substrates involved in perceptual shifts and/or training effects reported here. Because of the simple, sinusoidal nature of SWS, it may also be possible to measure simultaneous stimulus frequency otoacoustic emissions in addition to neural responses from the brainstem and cortex. Such an approach would allow for context or training effects to be studied from cochlea to cortex using the same stimuli.

## Data Availability Statement

The raw data supporting the conclusions of this article will be made available by the authors, without undue reservation.

## Ethics Statement

The studies involving human participants were reviewed and approved by the University of Texas at Austin IRB. The patients/participants provided their written informed consent to participate in this study.

## Author Contributions

All authors listed have made a substantial, direct, and intellectual contribution to the work, and approved it for publication.

## Conflict of Interest

The authors declare that the research was conducted in the absence of any commercial or financial relationships that could be construed as a potential conflict of interest.

## Publisher’s Note

All claims expressed in this article are solely those of the authors and do not necessarily represent those of their affiliated organizations, or those of the publisher, the editors and the reviewers. Any product that may be evaluated in this article, or claim that may be made by its manufacturer, is not guaranteed or endorsed by the publisher.
